# Digitizing tools for post introduction evaluation of rotavirus vaccine introduction in India

**DOI:** 10.1016/j.jvacx.2024.100502

**Published:** 2024-05-23

**Authors:** Pawan Kumar, Amanjot Kaur, Arindam Ray, Kapil Singh, Shipra Verma, Rhythm Hora, Seema S Koshal, Amrita Kumari, Rashmi Mehra, Syed F Quadri, Arup Deb Roy

**Affiliations:** a Immunization Division, Ministry of Health and Family Welfare, Government of India, India; b John Snow India, Delhi, India; c Bill and Melinda Gates Foundation, Delhi, India

**Keywords:** New Vaccine Introduction, Digitization, Post Introduction Evaluation

## Abstract

**Background and aims:**

The Rotavirus vaccine (RVV) introduction is a landmark event in the history of Indian public health as for the first time a novel, low-cost indigenous vaccine was introduced in a short timeline between 2016 and 2019. As per WHO mandate, post-introduction evaluation (PIE) be conducted within 6 to 12 months of vaccine introduction to provide an understanding of the operational aspects of the program. For RVV PIE, an innovative approach to developing and deploying a digitized tool was employed. The present study aims to document the processes followed for digitizing the data collection and analysis tools.

**Methods:**

The development of the RVV-PIE digital tool was undertaken in two phases. In the first phase, conceptualization and iteration of the modified WHO PIE tool were undertaken. Questions were organized sequentially to ensure natural progression in responses. The finalized questionnaire was converted to a digital version and extensive dummy data was entered to improve automated qualitative data analysis. Phase 2 involved updating the draft tool and incorporating changes to provide a field-tested version for deployment.

**Results:**

The digital version of the tool was successfully developed. The GPS functionality of the tool allowed live tracking of data collection making the process more accountable. The tool was prepopulated with reference materials and data points for easy reference and retrieval by the evaluators. The digitization of the tool also allowed easy visualization of data through maps, charts, and graphs on a real-time user-friendly dashboard.

**Conclusions:**

The digitization of the PIE tool for RVV in India has been a great learning experience where the dire situation of an ongoing pandemic catapulted us towards a more efficient and comprehensive process innovation. The RVV PIE tool could serve as a customizable digital PIE tool for other health programs heralding an era of a more effective and proficient process of PIE.

## Introduction

In 2016, India became one of the first countries in Asia to introduce a rotavirus vaccine, developed indigenously in the country [1], [2] under the Universal Immunization Programme and also the first country in the World Health Organization (WHO) South East Asia Region (SEAR) to do so [3]. Rotavirus vaccine (RVV) was introduced in a phased manner across the country from 2016 to 2019, including a rapid scale-up in 25 states and union territories (UTs) covering a cohort of around 12.1 million in 2019 under a “100-day agenda” of the Ministry of Health and Family Welfare (MoHFW), Government of India (GoI).

As per WHO’s recommendations, any new vaccine introduction must be followed by a Post Introduction Evaluation (PIE) within 6–12 months of the introduction to evaluate the overall impact of the new vaccine introduction on the National Immunization Programme [4], [5], [6], [7]. Due to the COVID-19 pandemic, the PIE was delayed and was conducted in March 2022. The objective of the RVV PIE was to assess the implementation process of the Rotavirus vaccine in the UIP and to capture the challenges, learnings, and best practices of rotavirus vaccine introduction and the RVV product switch in select states.

### Rationale for digitizing the RVV PIE Tool

The COVID-19 pandemic necessitated the need for digitizing the process of data collection and analysis to minimize physical contact and the spread of infection. The digital PIE tool was conceptualized to create a one-stop solution for multiple functions to reduce manual efforts and increase efficiency, as well as protect data loss in manual translation and transition. For the first time in India, the PIE was conducted using an innovative interactive digital tool. From the limited resources on PIEs conducted in other countries [8], [9], it was evident that these were conducted using traditional pen and paper-based tools. Hence, the present research centers on assessing the rationales and incentives for adopting a digital approach in conducting the RVV PIE. It explores the advancements offered by the digitized tool in comparison to the traditional pen-and-paper-based method [10], [11], [12]. It also delves into the challenges encountered at each stage, as well as the insights gained from utilizing the RVV PIE tool in India.

## Methodology

For the development of the RVV PIE tool, an external agency with support from the lead technical team at John Snow India Pvt Ltd was involved. The tool development process included due approvals from the Immunization Division, Ministry of Health and Family Welfare, Government of India. The state immunization officers were also consulted during the field testing and rollout of the online PIE tool.

### The study was conducted in different phases:

Phase I: Overseeing and driving the different stages of development of the digitized tool, dry run, collation of the feedback and challenges faced during the dry run, brainstorming for mitigation of these challenges, and finally, operationalizing the final tool during the actual data collection. The digitization process of the classical pen-and-paper questionnaires into the digitized survey tool took around 3 months. The process included changes in the storyline, number and sequence of questions at all levels. Then the pre-available data was linked to the respective questions for all visited states and districts. This inclusive involvement of the team in every step facilitated the collation of continuous ideas and thoughts and prevented the loss of any ideas on the way.

Phase II: A comprehensive desk review was done to collate the already published advantages and challenges of using digital tools in comparison to the conventional pen-and-paper method. A literature search was conducted through different databases, including Google Scholar, PubMed, EbeSco, and CINAHL for the publications during the last fifteen years. The topics included in the search were: new vaccine introduction, Rotavirus vaccine introduction, digital data collection, digital tool advantages in health surveys, time-saving in data collection, data accuracy in health surveys, geo-tagging in health surveys, data storage and retrieval in vaccination studies, post introduction evaluation and real-time monitoring of vaccination surveys. Only those articles which were published in the last 15 years were included. The feedback and opinions gathered from all these resources were summarized through thematic content analysis.

### Results & Discussion

Based on the literature review and experience with rolling out the digital tool in the field, the following themes have emerged for discussion in this article: the innovation process for the development of RVV PIE, the factors driving the development of the tool, and the characteristic features of the tool that enhanced the evaluation process and experience of the evaluators.

A) Innovation process for RVV PIE

Table 1 summarizes the innovations that were designed and used for multiple steps in the RVV PIE, from data collection and tool development to data visualization.

**Table 1:** Comparison between conventional methods and the innovations done.

Process/Stage	Conventional Method	Innovation
Development of Survey Tool	• Large format questionnaires consisting of close to 100 questions • More factual questions than probing ones	• Questionnaire in story format for keeping the respondent engaged • Mix of subjective and objective questions with multiple choice options to enable ease of administration
Mode of recording responses	• Paper-based format for recording answers manually • Manual copies of instructions and reference material	• Standardized survey platform scripted with questions across all levels • Multiple features to ensure error-free recording of answers with ease
Monitoring of Survey	• Manual monitoring of the survey • Complex submission and collation process for filled forms	• Ability to monitor respondent-wise and level-wise completion status • Inbuilt features to ensure submission of completed forms only
Data collation and analysis	• Manual transcription of data to digital software like Excel, which is time-consuming • Manual cleaning and analysis of all qualitative and quantitative data	• Automatic recording of data in a standardized master template which saves effort for transcription, without errors • Automatic analysis of certain KPIs on the dashboard for both qualitative and quantitative data
Data visualization and reporting	• Manual collation of data for the creation of various graphs for KPI for reporting like PPTs, documentation, etc.	• Automatic visualization of selected KPIs providing a comprehensive view of KPIs on the same platform • Multiple filters enabling diverse visualization as per need

### Motivation to go digital for data collection

1. **Data Quality:** The primary reason to choose a digital data collection tool is that the features can be in-built for data quality checks [13].
a) **Skip patterns:** The digital platform had an embedded skip logic that helped save time and made it more efficient and user-friendly. It has been seen earlier that paper tools with frequent skip patterns were largely dependent on the investigator’s skills and knowledge and also the keenness to fill the form accurately. Hence, the paper tool was prone to human errors.
b) **Entry limits:** The ‘entry limit’ feature is important for the questions to which the answers are numeric and only the values between certain limits are to be involved or accepted for analysis. The entry limit feature saved time for the investigator and prompted him/her to not proceed with the rest of the questionnaire with that interviewee.
c) **Question types:** The digitized tool allowed the user to enter any answer format, including numeric, text, alpha-numeric, date, etc, as per the requirement of the question. The probability of errors introduced in the analysis and results was reduced.
d) **Optional vs mandatory questions:** The digitized tool automatically rendered questions as optional or mandatory, thereby removing the collection of incomplete or irrelevant data.
2. **Time saving:** Using a digital tool for data collection made the process of data collection efficient and time-saving [13], [14]. Time-saving features were added at several steps in the entire process.

First, the questions were designed in such a manner that all possible answers for any particular question have been thought of and put up as options from which the correct one can be selected and also having an ‘other’ option to elaborate any answer that has not been captured in the mentioned options. This step saves the time otherwise consumed while writing the entire sentence or answer by the investigator in a conventional pen-paper-based tool.

Second, if a pen-paper-based tool is used for data collection, the entire data set has to be transferred to an Excel sheet to collate data from different entry points, clean, and analyze the same. This data transfer is time-consuming and error-prone. Also, data cleaning and data visualization are time-intensive.

On the other hand, if a digitized tool is used, which has pre-identified KPIs, data entry points identified for each visualization, and a backend analysis automation done, all these steps can be cut and the data entry from all devices at different survey locations can be the only step required as the backend functions will do the rest. A dashboard can be attached with these KPIs where a first-cut analysis with different visualizations can be created automatically.

3. **Data Accuracy:** The use of digitized tools helps minimize data collation errors and thus the quality of data analysis is improved. For the pen and paper method, several potential error points were mapped.
o While reading the answers from the paper if the person entering the data is different than the one who actually wrote the answers in the survey, the writing might be intelligible, or a shorthand used which the data entry personnel might not understand, and decide not to ask the investigator.
o Data entry errors can occur inadvertently while typing onto the digital platform.
o While collating data from different users, onto one platform, a few cells/columns/rows can be missed while copying and pasting from one file to another.
o The data entry may be incomplete.
o Similarly, errors can occur while manually selecting fields for developing the KPIs.
4. **Reference data reports:** For surveys like the Post Introduction Evaluation, a lot of the data of the said vaccine in particular and the Routine Immunization Programme in general has to be taken along by the investigators, as a point of reference for a few data based questions in the tools. Earlier, all these data reports, which might be in the forms of graphs, charts, color-coded coverage maps, etc., were printed on paper, and carried along by the investigators. These formed a huge paper load, small and large, which could be misplaced, damaged, or not legible. Again, finding the particular reference data paper concerning the question being filled from a huge pile of papers was a challenge.

Using a customized digitized tool, on the other hand, had all these data references added as a pop-up at the respective questions. Also, the data of that particular region/area came in the tool of particular investigators only (not for all states and districts from which the investigator had to find the relevant data set). This helped remove the long paper trail and time of the investigator.

**Fig. 1:**
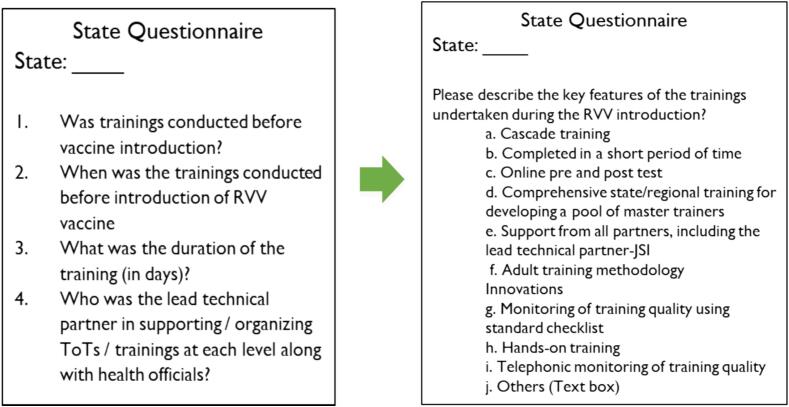
Sample of a question drafted for the digital RVV PIE tool.

5. **Clutter-free and hassle-free:** If paper-pen tools were used, in addition to the reference material to be provided to each investigator, the entire tools had to be printed, stationary bought and provided to each investigator. Though there were no direct costs associated with data entry in this methodology, plenty of personnel time was saved as this entire process could be quite tiresome and time-consuming compared with the digital data collection tool. With the use of a digitized tool, all such efforts were not required as the investigators only had to log in to the hand held device (tablets) to access the required questionnaires particular to the region they were visiting.
6. **Data storage and retrieval:** Data collection using a conventional paper-pen method being time-consuming, cumbersome and difficult is also not very feasible or practical for data storage and retrieval. Keeping stacks of paper carefully, without them being damaged by liquids, fire, etc. accidentally or the writing being smudged or the papers torn is a task in itself. The pages on which the data has been collected might be damaged due to several reasons and if so, the retrieval of that data would be impossible or difficult at most. Also, keeping these stacks of paper requires a lot of storage space as well. Whereas the digitized tool used was programmed to save the data automatically on the cloud, whether the user was online or offline, preventing loss of any data. As the data was stored with date and time stamps along with the place of data recorded (if GPS was enabled), data retrieval was also easily done [13], [15], [16].
7. **Environment friendly:** Today, environmental issues are a sensitive and hot topic everywhere. Keeping that in mind, using a digitized tool for data collection was an added plus saving the use of loads of paper coming from cutting trees [13], [15], [16]. The less the paper is used, the more beneficial for the environment.
8. **Geo-tagging of the surveyors (capturing GPS locations):** Many times it so happens that the investigators make mental notes to be entered after returning to the hotel and this may lead to errors in capturing data. Recent developments and innovations have enabled geo-tagging of the digital data collection devices’ real-time location. This ensures that the investigators have entered the data while at the session site, the cold chain point or during house-to-house surveys [15]. This step again ensured accurate data entry.
9. **Preventing data loss:** Using the paper tools, translation, and transition of the data from paper to the analysis tool, there can be loss or misinterpretation of data. When we customized and pre-coded the data collection tool, as soon as the data was entered and saved, it automatically got transferred to the analytical tool. Therefore, there was no possibility of any data loss [13], [14], [17].
10. **Real-time robust monitoring of the tool:** The digital tool had a completion dashboard, which showed the team-wise completion status [13], [16], [17]. This was visible to the central control room members and this enabled the central team to reach out to the team/s that were a bit slower in data collection compared to the majority. Thus, the challenges encountered were addressed and data collection was expedited.
B) Development of the Digital tool:

For the first time, a digital comprehensive tool for a PIE, across the world for any vaccine, was developed. The digital tool was developed from scratch, with the WHO standard PIE tool taken as the basis for drafting the RVV PIE tool. The standard tool consisted of large questionnaires, around 90–100 questions, all of which were single-point questions with straight answers. A lot of the factual questions in the standard questionnaire were deemed redundant from an evaluation standpoint and could have been converted into a multiple-option single question. Also, instead of single-standing questions, the RVV PIE tool was designed in a story format to maintain short lengths of each level questionnaire. This also worked towards keeping the respondents engaged. The shortened questionnaires lead to the capturing of concise information, enabling analysis of only relevant answers Fig. 1.

A pool of public health specialists was involved in drafting the tools, which aided in designing crisp questionnaires where subjective questions were converted into well-researched objective questions with multiple-choice options. The tools were finalized after multiple deliberations with the Ministry of Health and Welfare and the digital tool was test run by the National Immunization officials before finalizing and making it live. This entire exercise took around 5 months before the tool was ready for field testing.

C) Features of the digital RVV PIE Tool

## General Features

The digital RVV PIE tool was hosted on a designated server and accessible via a link. Upon logging in, users were prompted to add the App to their device’s home screen, enabling offline access. The tool’s compatibility extended to smartphones, tablets, and laptops. Users from various states were assigned specific login credentials. While the initial login required an internet connection, subsequent usage allowed offline mode. Users were instructed to employ the same device for the entire survey, comprising all 6 questionnaires, saving drafts offline and submitting once back online. The platform offered flexibility to save responses during intermittent connectivity. Once submitted, response modifications were only possible if the central team unlocked the response. Upon tool closure, users could resume from where they left off, provided they had saved the responses. Data was cached on the device in both offline and online modes, with ‘submit’ transferring data to the cloud database. The questionnaires featured built-in pagination, displaying one question at a time with numbered color-changing bubbles indicating progress. Questions were categorized into predefined thematic areas, filterable through the ‘Theme’ dropdown.

## Special Features

**Fig. 2:**
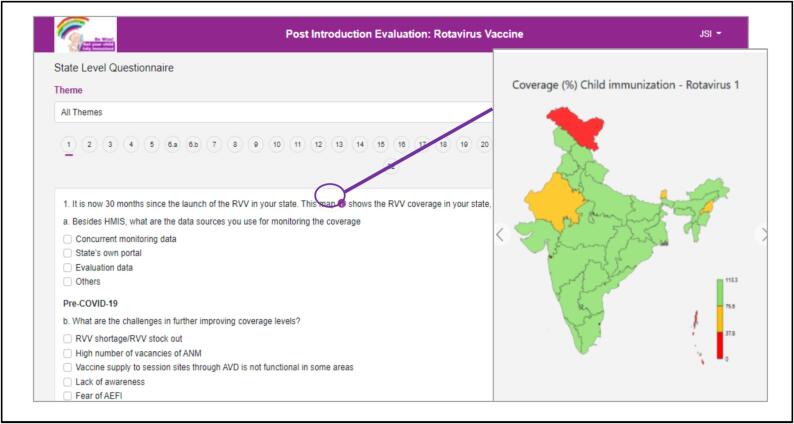
Pre-populated data in the tool.

**Fig. 3:**
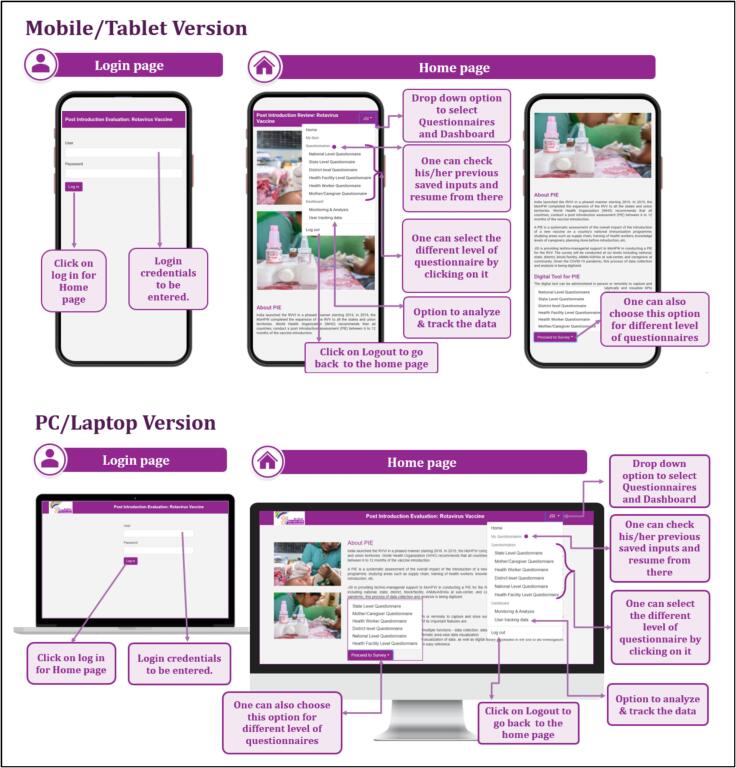
User ID and Password; Landing page of the tool: Mobile or Tablet Version and PC or Laptop version.

**Fig. 4:**
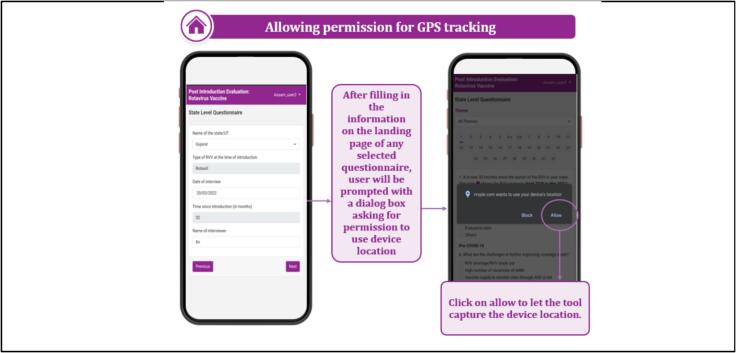
GPS tracking permission for device.

**Fig. 5:**
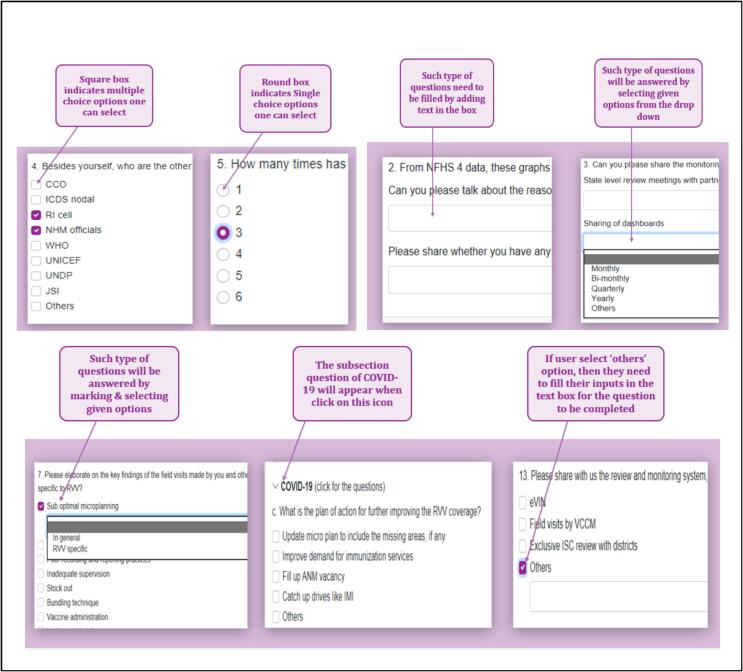
Different icon designs for different answer possibilities and In-depth detail prompts for a certain answer options.

**Fig. 6:**
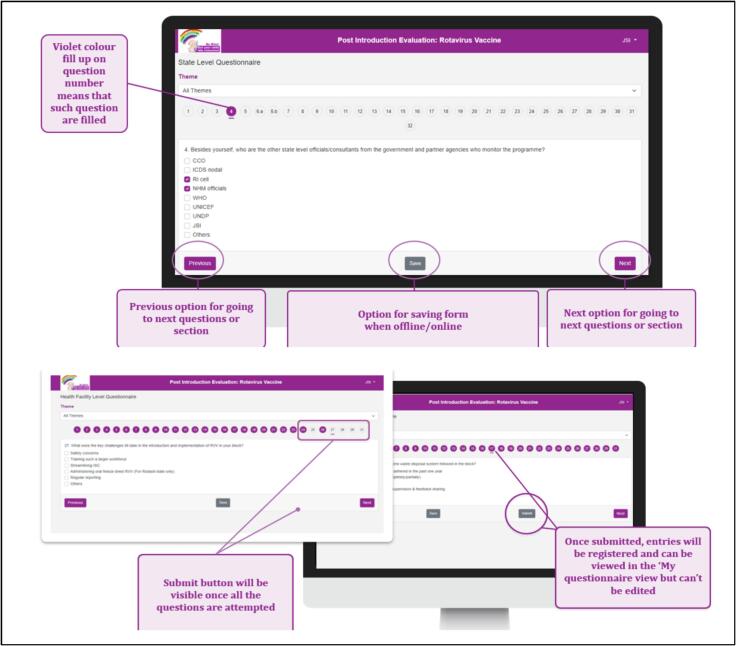
Question-completion feature of the tool; submission of the tool.

**Fig. 7:**
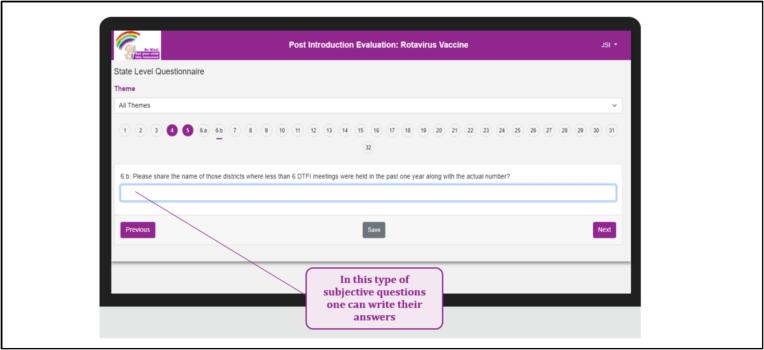
Subjective question’s feature in the tool.

The tool incorporated pre-populated reference charts and maps (Fig. 2) to elucidate key indicators about specific questions, aiding investigators in establishing context during interviews. To ensure accessibility, the tool was developed in multiple versions (Fig. 3), facilitating usage across different devices and serving as a backup utility. Upon logging in and selecting the questionnaire form level, a pop-up sought permission for GPS tracking (Fig. 4). The tool featured close-ended questions with distinct icons for single or multiple correct answers, while some questions prompted further exploration even after selection, requiring additional details for completion (Fig. 5). To address skipped questions, a color-coding system marked completed sections, allowing submission only when all questions were marked as complete (Fig. 6). Additionally, for questions necessitating descriptive answers, designated spaces were provided (Fig. 7).

## RVV PIE tool Dashboards

### Tool completion dashboard

**Fig. 8:**
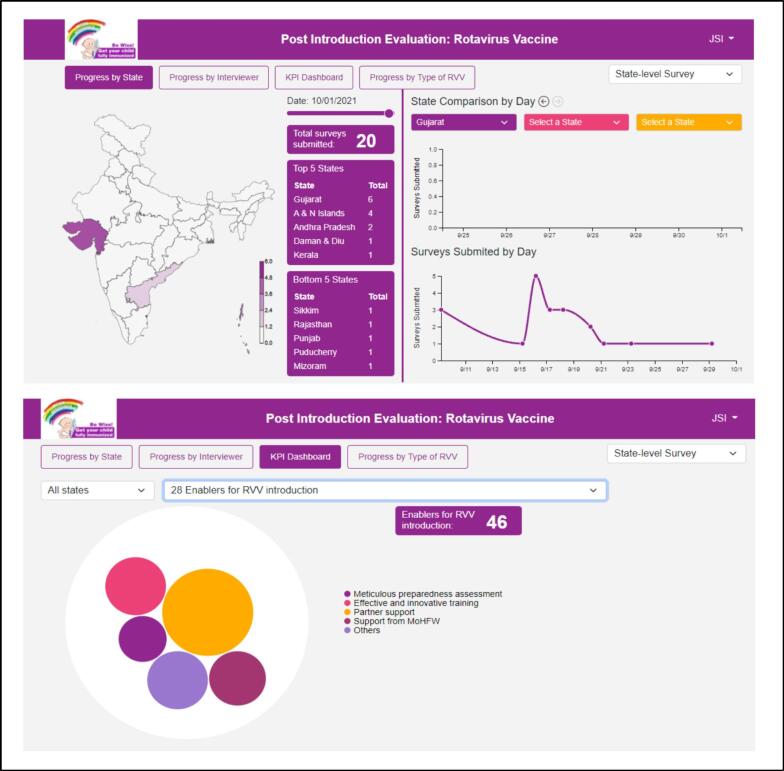
Dashboards: Tool completion and Data Visualization.

The utilization of a manual paper-based tool cannot inherently monitor tool completion and timeliness effectively. In contrast, the digitized tool provided a solution to this challenge. It enabled real-time monitoring of surveys across all levels through a unified platform, namely the tool completion dashboard. This digital system allowed for day-to-day tracking of survey progress, facilitating the monitoring of survey-related key performance indicators (KPIs) that could be harnessed as documented evidence. Fig. 8

A notable advantage of the digitized approach was the seamless management of responses, both online and offline. The system efficiently merged data collected from both modes into a single database. To ensure controlled oversight, a login-based monitoring dashboard was implemented, featuring varying levels of access for different user roles.

The implementation of real-time monitoring throughout the survey process fostered cohesive coordination among diverse teams operating in distinct geographical regions within the country. This digital solution not only addressed the limitations associated with manual tools but also revolutionized the monitoring and management of survey activities, enhancing efficiency and data quality.

### Data Collection, analysis, and visualization dashboard (Fig. 8)

Within the digitized PIE tool, a robust framework was established to facilitate data cleaning and real-time analysis, particularly focusing on quantitative data. Concurrently, the synthesis and analysis of qualitative analysis were advanced using metadata tags and natural language processing techniques. The tool harnessed TF-IDF (Term Frequency – Inverse Document Frequency) to underpin this process for the PIE survey, which has been in use for qualitative data analysis in recent years [18], [19], [20].

Investigators gained access to a survey analytics tab via a log-in system. Notably, the tool provided visual representations of all key performance indicators (KPIs)through various formats like geo-visualization, column charts, Gantt charts, and sunburst charts. The platform also allowed for the convenient download of KPI-specific data in Excel format, facilitating offline analysis and reporting.

This synchronization of functionalities ensured secure data transmission to the cloud database, minimizing the risk of loss. Furthermore, the tool empowered dynamic and diverse visualization for crucial KPIs, enabling the interpretation of responses across stakeholder levels. These findings could be shared with state managers as preliminary results, reinforcing transparency and collaboration.

**Fig. 9:**
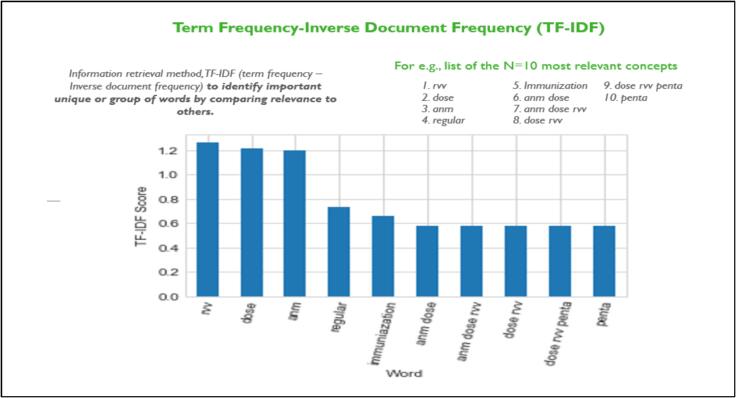
Term Frequency-Inverse Document Frequency (TF-IDF).

For questions featuring subjective answers, the integration of Term Frequency-Inverse Document Frequency (TF-IDF) scores was pivotal. This mechanism quantified the presence of predefined terms within responses, contributing to a structured assessment of qualitative data (Fig. 9). In essence, the digitized PIE tool not only streamlined data analysis and visualization but also facilitated secure data management and insightful interpretation.

### Challenges and Learnings

The RVV PIE in India was a landmark event in terms that for the first time a Post Introduction Evaluation was done on a large scale, across 14 states in the country, using a newly built digitized data collection, analysis and visualizations, and survey monitoring tool for the first time. Several challenges were experienced during different stages, i.e. the tool development, dummy run of the tool, field test, actual data collection, and the data analysis.

The first level of testing of the digitized tool was done during the dummy run. In the dummy run, the first drafted tool was tested by the programme implementation team to check for any tool functionality issues. Here, only seamless transit from one question to the next was seen, and that the submitted answers were recorded and saved at the backend.

The second level of testing was done in the field test. The field test was conducted to check the feasibility of the questions, appropriateness of the answer options, the functionality of tabs, time taken to complete each level of the questionnaire, and collection and short analysis of the responses collected to see whether the desired data and results were being derived. The field test was conducted in 4 districts across 4 states. A two-member team was sent to each of the selected four states for the field testing, and undertook the interviews in four days per state. Recommendations from the field test, shared by the state and district officials, were incorporated into the questionnaire which encompassed the inclusion of separate sections/questions on disease surveillance and safety surveillance. As gauged from the field exercise, the tool was perceived as a valuable and time-saving innovation for a large-level data collection activity. This exercise also aided in planning how the interviewers would be trained during the orientation of the PIE and national debriefings.

Due to these repeated tests done at different levels of digitization, challenges faced during the actual PIE survey were limited to difficulty in uploading real-time images and submitting the finalized surveys.

Based on the learnings from the challenges faced we would recommend a three-pronged approach:

First, an extensive dummy exercise using the tool in the field is imperative for checking the automated qualitative data quality. Next, tool validation before the actual survey should be carried out to see if the data culled out after collection is usable and easy to analyze. Last but not least, a comprehensive sensitization of the evaluators on how best to use the tool should be carried out before the survey, to familiarize them with the technology.

## Conclusion

The survey which requires a large amount of data collection in a short time and analytical and monitoring reports in even shorter times, digital data collection synched with the automation tool are the best solution for the investigators as well as for the program managers. The digitized tool used for the Post Introduction Evaluation of the Rotavirus vaccine in India can be modified and adapted for other PIEs within the country and can also act as a reference or base tool for other countries, for new vaccine introductions PIE.

The basic idea of this tool can be adopted for different surveys like preparedness assessments, process documentation and impact evaluations. It can also be used as evidence for advocacy on areas of improvement as all the data was collated and analyzed real-time that could be shared with the program managers at the time of the survey. Such digitized and automated tools can provide greater integration between qualitative and quantitative techniques when used for larger surveys.

This work serves as a valuable reference document for comparable surveys and Post Introduction Evaluations concerning new vaccines, both in India and globally, for those seeking to transition to digital methodologies.

## CRediT authorship contribution statement

**Pawan Kumar:** Writing – review & editing, Validation. **Amanjot Kaur:** Writing – original draft, Conceptualization. **Arindam Ray:** Methodology, Formal analysis. **Kapil Singh:** Visualization, Resources. **Shipra Verma:** Project administration, Methodology. **Rhythm Hora:** Visualization, Resources. **Seema S Koshal:** . **Amrita Kumari:** Writing – review & editing. **Rashmi Mehra:** Methodology. **Syed F Quadri:** Project administration. **Arup Deb Roy:** Writing – review & editing, Project administration, Conceptualization.

## Declaration of competing interest

The authors declare the following financial interests/personal relationships which may be considered as potential competing interests: [Amanjot Kaur reports writing assistance was provided by Bill & Melinda Gates Foundation].

## Data Availability

No data was used for the research described in the article.
